# Pacing cessation just before generator replacement - A life-threatening consequence of pacemaker battery depletion: A case report

**DOI:** 10.1016/j.jccase.2026.04.005

**Published:** 2026-05-07

**Authors:** Ryoto Mitsumizo, Yusuke Takahara, Hiroshi Ikuta, Ryota Komoto, Ryuichi Matsukawa, Keita Inanaga, Jun-ichiro Nishi

**Affiliations:** a Clinical Training Center, Aso Iizuka Hospital, Japan; b Department of Cardiology, Aso Iizuka Hospital, Japan; c Department of Clinical Engineering, Aso Iizuka Hospital, Japan

**Keywords:** Pacemaker, Pacemaker battery depletion, Elective replacement indication, End-of-life, Remote monitoring, Routine follow up

## Abstract

We report the case of a 77-year-old man who underwent single-chamber pacemaker implantation 16 years prior. The patient, who did not utilize remote monitoring, was lost to follow-up 14 years after the implantation, leading to profound battery depletion. This lack of surveillance resulted in a critical reduction of the pacing rate to 14 ppm, culminating in the complete cessation of pacing immediately before generator replacement. Despite the Abbott device's specified End-of-Life (EOL) safety mechanism, pacing function failed entirely—a rare but life-threatening complication. Uniquely, this case provides serial electrocardiographic documentation capturing the full progression of severe battery depletion. These findings underscore that continuous follow-up and remote monitoring, as emphasized in recent guidelines, are pivotal for preventing such critical outcomes.

**Learning objective:**

This case highlights the potential for profound pacemaker rate reduction and complete pacing cessation beyond manufacturer-defined End-of-Life settings in the setting of extreme battery depletion. It emphasizes the importance of regular device follow-up, timely generator replacement, and the use of remote monitoring to detect critical battery status changes and prevent life-threatening complications during pacemaker management.

## Introduction

Pacemaker batteries deplete in a predictable sequence, progressing from the Elective Replacement Indicator (ERI) to the End-of-Life (EOL) phase. Upon reaching EOL, most devices are programmed to revert to a fixed safety pacing rate to conserve remaining power, though this rate varies by manufacturer [1]. Delaying generator replacement beyond the EOL stage significantly heightens the risk of further functional decline, potentially leading to catastrophic clinical consequences [2]. While EOL pacing at a specified fixed rate is well documented [1], [2], instances of severe battery depletion causing the pacing rate to drop significantly below the standard EOL threshold are rarely reported. We present a case of a patient exhibiting VOO pacing at an extreme rate of 14 ppm, followed by the complete cessation of pacing just prior to generator replacement. This entire progression was captured through serial electrocardiograms (ECGs). This case highlights the critical dangers of neglecting pacemaker follow-up and underscores the essential role of remote monitoring in preventing life-threatening device failure.

## Case report

A 77-year-old man with symptomatic bradycardic atrial fibrillation underwent single-chamber pacemaker implantation (St. Emprise™ SR+ 5628, St. Jude Medical/Abbott; VVI 60 ppm) 16 years prior at a local hospital. The device was later reprogrammed to VVI 70 bpm. He had been followed up at a local clinic without remote monitoring until two years prior to the current presentation, when he visited our emergency department with acute-on-chronic heart failure. At that time, pacing was stable at 70 bpm. However, he subsequently declined a scheduled follow-up and failed to attend any further medical visits.

Ten days before the most recent admission, he developed progressive fatigue, anorexia, and weight gain, leading to a referral from a local clinic for suspected acute heart failure. Upon arrival, the patient's vital signs were blood pressure 160/73 mmHg, heart rate 50/min, respiratory rate 20/min, and SpO2 97% (room air). Physical examination revealed marked lower-extremity edema and audible wheezing. Laboratory tests revealed a B-type natriuretic peptide (BNP) level of 105 pg/mL, and chest radiography showed a cardiothoracic ratio of 62%. The admission ECG showed VOO pacing at a severely reduced rate of 24 ppm (pacing interval: 2480 ms) with escape beats at 40 bpm. Device interrogation was deferred due to the strong suspicion of severe pacemaker battery depletion. Emergent generator replacement was advised due to the risk of life-threatening arrhythmia; however, the patient insisted on next-day admission despite counseling on the risk of sudden death.

On the following day, the patient returned with worsening dyspnea. Upon arrival, his vital signs were: blood pressure 151/54 mmHg, respiratory rate 20/min, and SpO2 95% on room air. The ECG revealed VOO pacing at an extremely low rate of 14 ppm (pacing interval: 4200 ms) accompanied by escape beats at 40 bpm (Fig. 1).

**Fig. 1:**
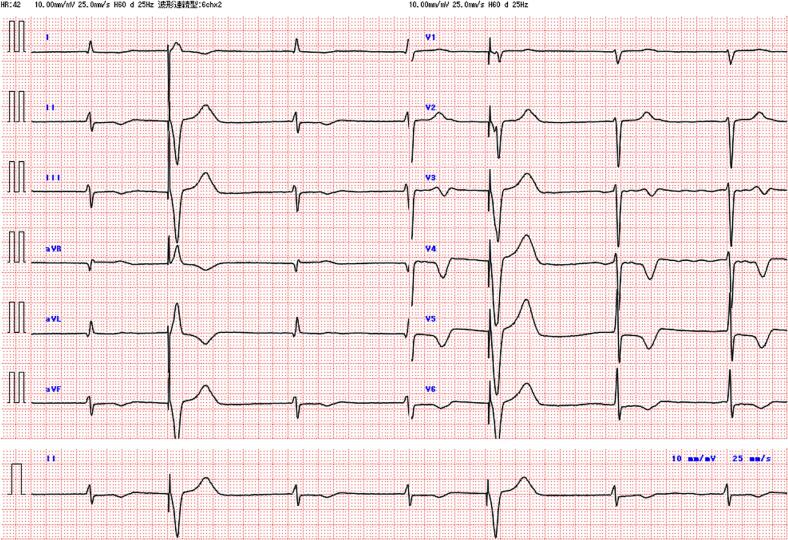
ECG on presentation to the emergency department. ECG revealed VOO pacing at 14 ppm (pacing interval: 4200 ms) with escape beats at 40 bpm.

Emergent generator replacement was performed. In accordance with our institutional policy, temporary transvenous pacemaker insertion was deferred because the patient maintained hemodynamic stability with a reliable intrinsic escape rhythm, and to minimize the risk of procedure-related infection. Instead, the patient was transferred to the operating room with transcutaneous pacing pads applied as a precautionary measure. A Medtronic PlasmaBlade X4.0™ was utilized for tissue dissection and hemostasis. Table 1 and Fig. 2A-G summarize the intraoperative events and electrocardiographic findings from the time of entry to the operating room until the completion of the procedure. Notably, the pacing interval progressively lengthened during the operation, culminating in the complete cessation of pacing just before the explantation of the depleted generator. No significant hemodynamic instability, including alterations in blood pressure or oxygen saturation, was observed during the period of pacing cessation. Connection of the device programmer restored stable pacing immediately. The old generator was replaced, and stable pacing at VVIR 70 ppm was confirmed before concluding the procedure. After optimization of his chronic heart failure therapy, the patient was discharged without further events. Remote monitoring was initiated, and the next visit to our outpatient clinic was scheduled after one year, with a local hospital designated to provide ongoing interim follow-up care.

**Table 1:** Timeline from presentation to the emergency department to generator replacement after transfer to the operating room.

Time	Event	Pacing interval	Figure
One day prior to admission	When visiting the emergency room.	2480 ms	–
Admission day 13:33	When the patient was transported to the hospital by ambulance the following day.	4200 ms	Fig. 1
14:50	The patient was brought into the operating room. VOO pacing with escape beats.	4520 ms	Fig. 2A
14:55	VOO pacing with escape beats.	4800 ms	Fig. 2B
14:58	VOO pacing with escape beats.	4600 ms	Fig. 2C
15:01	VOO pacing with escape beats.	5120 ms	Fig. 2D
15:16	VOO pacing with escape beats.	6480 ms	Fig. 2E
15:20	No pacing spikes can be observed.	No pace	Fig. 2F
15:23	The generator was replaced.	No pace	–
15:24	After connecting to the device programmer, stable pacing was achieved.	1320 ms	Fig. 2G

**Fig. 2:**
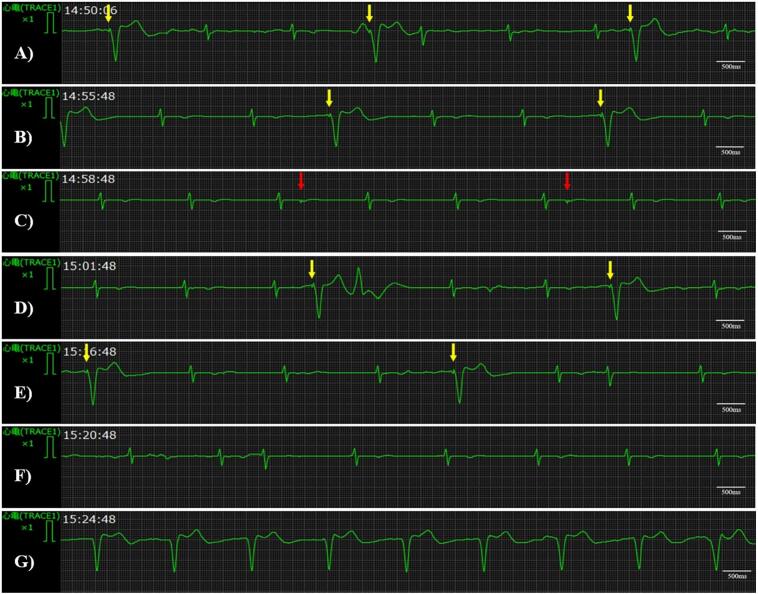
ECG after transfer to the operating room. A) Lead II electrocardiogram at 14:50. ECG revealed VOO pacing (pacing interval: 4520 ms) with escape beats at 41 bpm. B) Lead II electrocardiogram at 14:55. ECG revealed VOO pacing (pacing interval: 4800 ms) with escape beats at 38 bpm. C) Lead II electrocardiogram at 14:58. ECG revealed VOO pacing (pacing interval: 4600 ms) with escape beats at 39 bpm. D) Lead II electrocardiogram at 15:01. ECG revealed VOO pacing (pacing interval: 5120 ms) with escape beats at 37 bpm. E) Lead II electrocardiogram at 15:16. ECG revealed VOO pacing (pacing interval: 6480 ms) with escape beats at 39 bpm. F) Lead II electrocardiogram at 15:20. ECG revealed escape beats at 39 bpm. No pacing spikes can be observed. G) Lead II electrocardiogram at 15:24. After connecting to the device programmer, ECG revealed stable VVI pacing at 45 bpm (pacing interval: 1320 ms). The yellow arrow indicates normal pacing, whereas the red arrow indicates pacing failure or pacing occurring during the refractory period.

## Discussion

In the Abbott device (St. Emprise™ SR+ 5628) used in this case, the EOL mode is characterized by the loss of rate responsiveness and a 100-ms extension of the pacing interval, resulting in a nominal safety pacing rate of approximately 67.5 ppm [1]. However, in this patient, the pacing rate dropped to an unprecedented 14 ppm—significantly below the specified EOL rate—indicating complete battery depletion far beyond the designed safety mode. While a transition to a fixed pacing rate due to battery depletion is widely recognized [1], [2], the unique aspect of this case is that the pacing function failed entirely just before generator replacement due to severe depletion, with the complete process documented by ECG.

In the present case, a stepwise reduction in the pacing rate was observed. To our knowledge, no previous reports have described the mechanism of a stepwise decrease in pacing rate associated with battery depletion. We therefore contacted the device manufacturer, Abbott; however, no definitive explanation was provided. Based on the technical comments received from the manufacturer, the following mechanism may be considered. In implantable pacing circuitry, stimulation pulses are generated by discharging a capacitor that has been charged by the battery. The time required to recharge the capacitor to the programmed voltage directly affects the pacing interval. As battery voltage declines and the available current decreases, the capacitor charging time becomes prolonged. Consequently, progression of battery depletion may lead to prolongation of the pacing interval, which can manifest clinically as a stepwise reduction in pacing rate.

Such an extreme rate drop and subsequent pacing failure carry a very high mortality risk. Sinha et al. [3] investigated the clinical symptoms of pacemaker battery depletion in patients at ERI status, reporting conditions such as heart failure, chest infection, pacemaker syndrome, presyncope, and palpitations. In the present case, the patient developed congestive heart failure directly attributed to pacing failure. As the pacemaker battery depletes, symptoms may progressively worsen, leading to syncope, which has been suggested to result not only from profound bradycardia but also from Torsade de Pointes [2]. Liu et al. reported a case of an 80-year-old woman in whom a pacemaker at EOL status developed undersensing, leading to R-on-T–induced torsade de pointes [2]. They emphasized that, when pacemaker recipients present with unexplained ventricular arrhythmias—particularly torsade de pointes—clinicians should consider the possibility of pacemaker battery depletion. In the present case, battery exhaustion resulted in undersensing and precipitated R-on-T events, as illustrated in Fig. 2. These findings strongly suggest that battery longevity constituted a functional risk factor contributing to the onset of a potentially fatal arrhythmic event. This emphasizes the need for rapid intervention in cases of severe battery depletion, especially when device function becomes unpredictable.

To prevent such catastrophic EOL states, adherence to regular pacemaker checks and the implementation of remote monitoring are crucial. The 2021 ESC Guidelines on Cardiac Pacing and Cardiac Resynchronization Therapy recommend pacemaker interrogation every 12 months for patients with single/dual-chamber pacemakers, or every 18 to 24 months when combined with remote monitoring. Although remote monitoring is a Class I recommendation for patients with difficulties attending in-office visits, its universal implementation remains Class IIa [4]. Similarly, Japanese guidelines recommend annual in-person programmer interrogation as Class I, while the routine use of remote monitoring for all patients is Class IIa [5]. The PONIENTE trial demonstrated that remote monitoring was as effective as in-hospital monitoring in elderly patients and significantly reduced hospital admissions by facilitating the early detection of both clinical and device-related adverse events [6]. In this case, the primary cause of the life-threatening battery depletion was the patient's non-adherence to follow-up appointments combined with the absence of remote monitoring. At discharge, we initiated remote monitoring and arranged regular outpatient follow-up. We therefore suggest that this approach may help reduce the rate of pacemaker-related rehospitalization.

## Conclusions

We report a rare and critical case of complete cessation of pacing immediately before generator replacement, caused by profound pacemaker battery depletion. This resulted in a pacing rate drastically below the standard EOL safety setting, with the entire process documented by serial ECG recordings. This case serves as a crucial reminder, highlighting the vital necessity of continuous device surveillance, robust patient education, and the proactive adoption of remote monitoring to prevent life-threatening device failures stemming from loss to follow-up.

## Consent statement

Written informed consent was obtained from the patient.

## Declaration of competing interest

We have no conflicts of interest.
